# Comparative analysis of expressed sequence tags from three castes and two life stages of the termite *Reticulitermes flavipes*

**DOI:** 10.1186/1471-2164-11-463

**Published:** 2010-08-06

**Authors:** Matthew M Steller, Srinivas Kambhampati, Doina Caragea

**Affiliations:** 1 Department of Entomology, Kansas State University, Manhattan, KS 66506, USA; 2 Department of Computing and Information Sciences, Kansas State University, Manhattan, KS 66506, USA

## Abstract

**Background:**

Termites (Isoptera) are eusocial insects whose colonies consist of morphologically and behaviorally specialized castes of sterile workers and soldiers, and reproductive alates. Previous studies on eusocial insects have indicated that caste differentiation and behavior are underlain by differential gene expression. Although much is known about gene expression in the honey bee, *Apis mellifera*, termites remain relatively understudied in this regard. Therefore, our objective was to assemble an expressed sequence tag (EST) data base for the eastern subterranean termite, *Reticulitermes flavipes*, for future gene expression studies.

**Results:**

Soldier, worker, and alate caste and two larval cDNA libraries were constructed, and approximately 15,000 randomly chosen clones were sequenced to compile an EST data base. Putative gene functions were assigned based on a BLASTX Swissprot search. Categorical *in silico *expression patterns for each library were compared using the R-statistic. A significant proportion of the ESTs of each caste and life stages had no significant similarity to those in existing data bases. All cDNA libraries, including those of non-reproductive worker and soldier castes, contained sequences with putative reproductive functions. Genes that showed a potential expression bias among castes included a putative antibacterial humoral response and translation elongation protein in soldiers and a chemosensory protein in alates.

**Conclusions:**

We have expanded upon the available sequences for *R. flavipes *and utilized an *in silico *method to compare gene expression in different castes of an eusocial insect. The *in silico *analysis allowed us to identify several genes which may be differentially expressed and involved in caste differences. These include a gene overrepresented in the alate cDNA library with a predicted function of neurotransmitter secretion or cholesterol absorption and a gene predicted to be involved in protein biosynthesis and ligase activity that was overrepresented in the late larval stage cDNA library. The EST data base and analyses reported here will be a valuable resource for future studies on the genomics of *R. flavipes *and other termites.

## Background

Examining the gene expression differences underlying social development and behavior has been termed sociogenomics [1]. Sociogenomics is predicated on two observations: "social life has a biological basis and thereby is influenced by genes and evolution" and "molecular functions of many genes are conserved across species" [1]. Therefore, it is possible to examine eusocial species such as termites, honey bees, ants, and social wasps in an ecological and molecular context to better understand the genetics, function, and origins of eusocial behavior.

Termites (Isoptera) are a large and diverse group of eusocial insects. Although eusociality is believed to have evolved only once in Isoptera, the ~2600 described species show enormous diversity in life history, behavior, colony composition, morphology, physiology, ecology, and biogeography [2,3]. Members of the genus *Reticulitermes *(Rhinotermitidae) are found throughout much of the contiguous United States, with introduced populations in Europe, South America, and the Bahamas [4]. *Reticulitermes flavipes *Kollar, the eastern subterranean termite, is the most common species in the United States. *R. flavipes *colonies, like those of other termites, consist of morphologically and behaviorally specialized castes. All individuals emerge from eggs as larvae, beyond which the development is flexible in that larvae may develop into nymphs and subsequently into dispersing alates, or into the sterile workers or soldiers [reviewed in ref. [2]]. Workers are involved in nest building, tunnel maintenance, and brood care whereas soldiers defend the colony and possibly communicate general colony fitness. Workers and nymphs of *R. flavipes *may develop into supplementary reproductives under certain conditions [2,3].

Historically, *Reticulitermes *spp. have been difficult to study mainly due to their cryptic living conditions and amorphous nest structure [2,5]. However, the advent of genomic and bioinformatic methods has provided new opportunities to study these organisms [2]. A macroarray study of *R. flavipes *[6] demonstrated differences in gene expression among the different castes. Genes that exhibited differential expression between workers and soldiers included salivary cellulases, endoglucanases, tubulins, and troponins [6].

Other examples of differential gene expression among termite castes include higher expression of the mitochondrial cytochrome oxidase-III subunit in workers and nymphs of *R. santonesis *[7], increased cytochrome P450 levels after methoprene application in soldier and pre-soldier fat body in *Hodotermopsis sjostedti *and *Nasutitermes takasagoensis *[8,9], and a gene specifically expressed in *H. japonica *soldier mandibles termed SOL1 thought to be involved in soldier specific behavior [10].

In the well-studied eusocial honey bee, *Apis mellifera*, there is a tendency for behaviorally associated genes to exhibit a G+C bias within promoter regions when compared to *Drosophila melanogaster *and more *cis*-regulatory elements [11] as well as high levels of expression of ribosomal and hexameric storage proteins in queen and worker cDNA libraries [12]. An EST analysis revealed that honey bee workers showed an over-expression of genes involved in cell differentiation and hydrolase activity, whereas honey bee queens exhibited an upregulation of genes involved in metabolism and oxidoreductase activity [13].

Given that there has been no large-scale effort to identify expressed genes in termites, our first objective was to characterize *R. flavipes *genes by analyzing an EST database of ~15,000 clones from three cDNA libraries constructed from workers, soldiers, and alates as well as two cDNA libraries constructed from early and late termite larval stages. Our second objective was to undertake an *in silico *analysis of expression patterns among the castes and life stages to tentatively identify putatively differentially expressed genes.

## Methods

### Termites

Five cDNA libraries were constructed from *R. flavipes *workers, soldiers, alates, and larval stages collected in March 2006 from a single colony on the campus of the University of Florida, Gainesville. The larvae were divided, based on size, into early (putatively stages 1-2) and late (putatively stages 3-4) stages. Several hundred individuals of each caste/stage totaling about 2 gm of wet weight were obtained and stored at -80°C until used. The individuals were not separated according to sex; therefore, the cDNA libraries contained expressed genes in both males and females, although we do not know the precise sex ratio among individuals that were used for mRNA isolation and cDNA synthesis. The larvae have a dichotomous developmental pathway, developing into either nymphs (which then become alates or second-form reproductives) or workers (some proportion of which subsequently become presoldiers and then soldiers).

### cDNA Libraries

mRNA isolation, cDNA synthesis and library construction was undertaken by AGCT Corp. (Cranston, RI). The cDNA was non-normalized and cloned into pBluescript II SK+ vector after digestion with *Eco*RV and *Not*l. We did not attempt to systematically exclude bacterial, protozoan, or other symbiont DNA from the libraries. Libraries were plated on LB medium with 50 mg/litre ampicillin and grown at 37°C overnight. Several hundred colonies were sampled randomly from the plates for further processing and sequencing. Colonies were placed in 150 μl liquid LB medium containing ampicillin, grown overnight at 37°C, and stored at -80°C after adding 20 μl of sterile glycerol. The colonies were then shipped on dry ice to the University of California-Riverside for sequencing the 5'-ends of the clones using the T3 primer.

### Sequence Analyses

Using Sequencher (v4.7, Gene Codes Corp, Ann Arbor, MI), we removed the vector sequence and trimmed ends using program defaults. Sequences shorter than 150 bp were discarded from further analysis after trimming. ESTs were organized into contiguous sequences (contigs) using the default parameters in EGassembler [14]. Highly repetitive sequences were masked using the *Drosophila *RepBase library in EGassembler.

Gene discovery rate for each library was estimated by dividing the number of contiguous sequences by the total number of singleton sequences. Minimum average sequence insert sizes of cDNA clones were estimated by dividing the total number of base pairs sequenced by the number of ESTs in each library. The maximum length is limited by the length of sequence; thus, the actual insert size was likely underestimated. Both singletons and contigs were assigned putative functions using a batch BLASTX reference search from the non-redundant protein database using BlastStation (v2.61, TMSoftware, Arcadia, CA), with an e-value ≤1e^-10^. Genes assigned putative housekeeping roles were identified using a list derived from Eisenberg et al. [15]. Gene ontology (GO) enrichment analysis was performed using Blast2GO tool [16]. The functional annotation in Blast2GO was performed in three steps. First, gene sequences were queried against gene or protein databases and potential homologs identified. During the mapping step, known GO annotations of the homologs were retrieved. Finally, the homolog annotations were used to annotate the uncharacterized genes.

For a set of annotated genes (including genes for which the annotations are obtained outside Blast2GO), several tools for statistical and visual analysis are available. Among these, Blast2GO includes a tool for performing enrichment analysis, i.e., the identification of GO annotations whose abundance is significantly different between two sets of annotated genes. The GO term enrichment analysis functionality is achieved by integrating Blast2GO with Gossip, a software package that employs Fisher's exact test to estimate the significance of associations between two categorical variables, while correcting for multiple testing using FDR (false discovery rate), FWER (family-wise error rate) and single test *p*-value. A set of GO terms that are under- or over- represented at a specified significance value is obtained as a result of performing the enrichment analysis.

All EST sequences are deposited in dbEST http://ncbi.nlm.nih.gov/dbEST/ under dbEST-Id 65538065-65551007, and in GenBank under accession numbers G0898823-G0911765.

### *In silico *transcript abundance

Relative transcript abundance in the ESTs derived from each of the five cDNA libraries was analyzed using the R-statistic [17]. The R-statistic can be seen as entropy of partitioning contigs (corresponding to genes) among multiple cDNA libraries, and can be used to identify differentially expressed genes as those genes with high R-value [17]. More precisely, the test statistic R compares the null hypothesis, L_0 _("the abundance of a transcript is the same in all libraries"), with the alternative hypothesis, L_1 _("the abundance of the transcript in each library is different", i.e., the corresponding gene is differentially expressed), using the log ratio of the two likelihoods, i.e., log(*L*_*1*_/*L*_*0*_). Thus, for a particular transcript (contig) *j*, we have:

$$\begin{array}{cc} R_{j}=\sum_{i=1}^{m}x_{i,j}\log\left(\frac{x_{i,j}}{N_{i}f_{j}}\right) & f_{j}=\frac{\sum_{i=1}^{m}x_{i,j}}{\sum_{i=1}^{m}N_{i}} \end{array}$$

where *m *is the number of EST libraries considered, *x*_*i,j *_is the number of ESTs in contig *j *belonging to the *i*th library, *N*_*i *_is the total number of ESTs in the *i*th library and *f*_*j *_is the frequency of all of the ESTs of contig *j *in all of the libraries. If *x*_*i,j *_= 0 for a library *i*, then the contribution of the *i*th library to *R*_*j *_is 0. Compared to other similar methods (e.g., Fisher's exact test), which can only work for two libraries at a time, the R-statistic can work with a multiple libraries. This feature makes the use of the R-statistic desirable for our analysis.

We analyzed the 20 contigs with the highest R-value, including 10 contigs that returned a BLASTX hit with an e-value ≤1e^-10 ^and 10 that had no BLAST hit or a hit with an e-value ≥1e^-10^. The cut-off for the number of contigs (genes) differentially expressed was identified by investigating the frequency of R-statistic among the contigs as described [17]. Putative protein functional analysis of the latter group (i.e., those contigs with no BLAST hit or e-value ≥1e^-10^) was performed using the protein function predictor [18] which pools sequences with similar structural motifs and assigns GO terms, to obtain an initial indication of gene function. The protein function predictor attempts to predict protein function to a large number of genes than a conventional BLAST search by providing low-resolution function without losing accuracy. We further validated the R-statistic measure of significant difference using a chi-squared analysis.

## Results

### General Library Analysis

We sequenced 4031 worker, 3836 alate, 3548 soldier, 2111 early larval stage, and 1733 late larval stage cDNA clones for a total of 15,259 clones. Each cDNA library had a minimum average insert size ranging from 570 to 1086 bp; the gene discovery rate ranged from 47% to 60% (Table 1). Each cDNA library had a 70-90% of genes with no related sequences in public data bases; of the remaining genes, the worker and late larval libraries had the highest proportion of ribosomal sequences and both larval libraries exhibited the highest level of sequence redundancy after BLASTX analysis (Table 1). Each library contained between 5% and 16% of sequences that had an e-value of ≤1e^-10 ^and were used in the GO analysis.

**Table 1:** Basic characteristics of the five cDNA libraries used in this study.

	Worker	Alate	Soldier	Early Larval	Late Larval
**No. clones sequenced**	4031	3836	3548	2111	1733

**Total <150 bp (discarded)**	1069	330	705	99	113

**Min. mean insert size (bp)^1 ^**	570	1086	638	681	416

**Gene discovery rate^2^**	60%	47%	58%	56%	51%

**Unigenes**	1787	1639	1637	1136	792

^1 ^Minimum mean insert size was calculated as the total number of basepairs (after sequence trimming, vector removal, and discarding of sequences less than 150 bp) divided by the number of clones sequenced.

^2 ^Gene discovery rate was calculated as the number of contigs divided by total number of clones sequenced.

### Sequence Analysis

**Figure 1:**
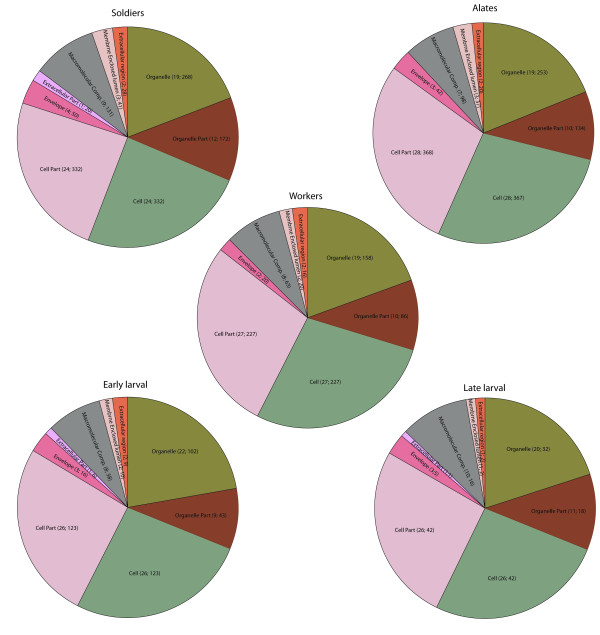
**Proportions of different GO cellular component terms (level two) in each *R. flavipes *cDNA library**. The caste/life stage for each cDNA library is indicated above the pie diagram. The numbers next to each GO term are the percent and number of genes that are in that category.

**Figure 2:**
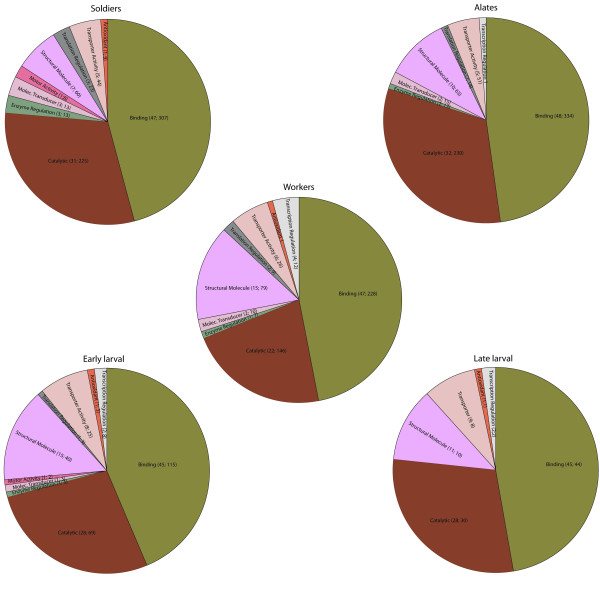
**Proportions of different GO biological process terms (level two) in each *R. flavipes *cDNA library**. The caste/life stage for each cDNA library is indicated above the pie diagram. The numbers next to each GO term are the percent and number of genes that are in that category.

**Figure 3:**
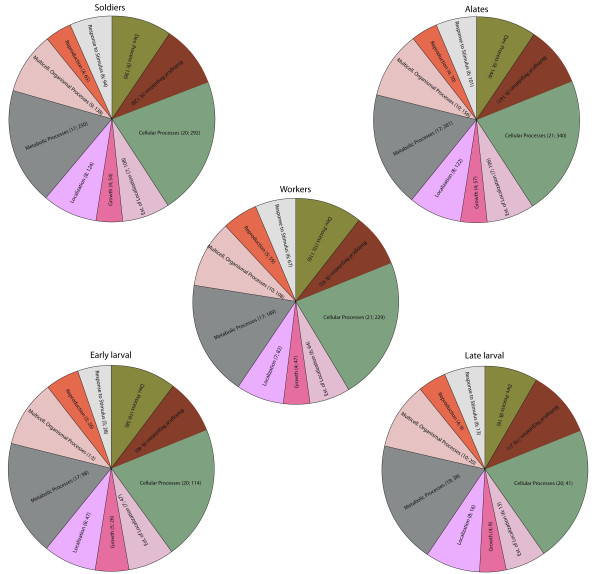
**Proportions of different GO molecular function terms (level two) in each *R. flavipes *cDNA library**. The caste/life stage for each cDNA library is indicated above the pie diagram. The numbers next to each GO term are the percent and number of genes that are in that category.

Analysis of the ESTs using level two cellular component GO putative terms showed similar proportions of the terms across castes with cell part, cell, and organelle, and organelle part being the most frequent terms assigned to the sequences (Figures 1, 2 and 3). The types and proportions of genes associated with the cellular component terms (Figure 1) were highly similar among the five cDNA libraries with minor differences in proportion. One difference was the absence of genes associated with extracellular part in alate and worker libraries. The GO analysis of level two molecular function terms showed that genes putatively involved in binding and catalytic activity represented the largest (>60%) proportion of ESTs in each library (Figure 2). In general, a greater variety of genes was detected in the solider, alate, worker, and early larval libraries relative to the late larval library. The third GO group we investigated was that of biological processes. Genes associated with ten different terms were detected in each of the five cDNA libraries in approximately equal proportions; those associated with cellular and metabolic processes were the most abundant in all five libraries (Figure 3).

**Figure 4:**
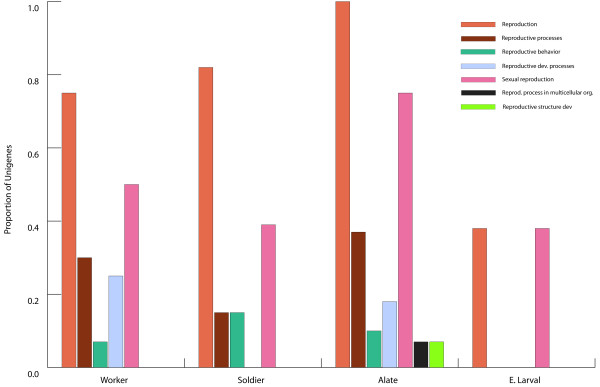
**Frequency of genes putatively matching reproductive GO terms in each *R. flavipes *EST library as a fraction to total number of unigenes**. No genes with putative reproductive functions were detected in the late larval cDNA library.

Several genes putatively associated with reproductive function were expressed in both sterile and non-sterile castes (Figure 4). Not surprisingly, the alates expressed the highest number and variety of transcripts (seven) associated with reproduction; however, the soldiers (five) and workers (four) also expressed a number of transcripts putatively associated with reproductive functions. Only two transcripts putatively associated with reproductive function were detected in the early larval stage cDNA library and none in the late larval stage cDNA library; the latter finding is not surprising considering we sequenced the fewest number of clones from the late larval stage cDNA library.

Due to the small number of BLASTX hits, we were unable to obtain many GO terms in proportion to the number of total ESTs to use in the statistical analysis of GO terms. Therefore, we chose a less stringent significance threshold than normally utilized with *p*-values ≤0.15; no significant differences in the proportion of GO terms were detected among the five cDNA libraries. Any differences described above must be interpreted with caution because of the random nature of the clone selection and the differences in the number of clones sequenced from each of the five cDNA libraries.

### *In Silico *Analysis of Genes With Significant BLAST Hits

**Figure 5:**
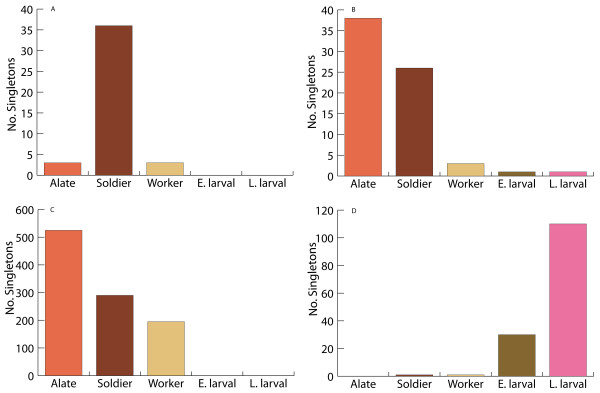
**A. R-statistic comparison of contigs composition and alate singleton bias**. (A) contig 794 of predicted putative ejaculatory bulb protein in soldiers; (B) contig 827 of predicted putative ejaculatory bulb protein in alates; (C) contig 545 of predicted neurotransmitter secretion or cholesterol absorption functioning in alates; (D) contig 366 of predicted protein biosynthesis or ligase activity functionality in late larval stage.

As mentioned, the contigs with significant R-statistic values were separated into two groups: those that returned a significant BLASTX hit and those that did not. For the first group (Table 2) the soldier library showed a bias of transcripts that putatively matched a protein from the cricket paralysis virus. Two separate contigs with an abundance in soldier and alate cDNA libraries showed different levels of sequence identity to the same ejaculatory bulb-specific protein in *Drosophila melanogaster *[Flybase:CG11390] (Figures 5A and 5B). Three other contigs showed a bias within the alate library that matched closely with cytochrome c oxidase (*Blattella germanica*), cellulase b (*Cellulomonas fimi*), and ATP synthase subunit a (*Drosophila simulans*). A number of contigs also showed overrepresentation in the early and late larval stage libraries including cytochrome b (*Drosophila sechellia*), tropomyosin (*Dermatophagoides farinae*), troponin c (*Tachypleus tridentatus*), cytochrome oxidase subunit 3 (*Drosophila melanogaster*), and 60 S ribosomal protein l31 (*Spodoptera frugiperda*).

**Table 2:** *In silico *analysis of transcript bias in contigs formed from all sequences in each library, the ten highest R-values were analyzed using BLASTX (e-value ≤1E^-10^).

Library Bias	Contig #	Length (bp)	**R**^**1**^	***p*-value**^**2**^	Identity	e-value	Identity (%)
Soldier	3	2647	106	5.24^e-50^	Viral Protein	1.00^e-170^	44

Soldier	794	1435	42	4.51^e-25^	Ejaculatory Bulb-specific Protein 3	1.00^e-25^	67

Alate	827	1036	31	1.42^e-10^	Ejaculatory Bulb-specific Protein 3	1.00^e-29^	73

Alate	148	981	66	6.63^e-21^	ATP Synthase Subunit A	1.00^e-27^	68

Alate	168	1724	26	1.03^e-10^	Cyctochrome C Oxidase Subunit 1	1.00^e-180^	86

Alate	352	1550	22	1.58^e-11^	Endoglucanase B Precursor (Cellulase B)	1.00^e-109^	59

Early Larval	472	954	92	3.61^e-48^	Cytochrome Oxidase Subunit 3	1.00^e-86^	76

Early Larval	479	1740	74	2.82^e-35^	Cytochrome B	1.00^e-134^	77

Early Larval	507	735	40	3.24^e-20^	Tropomyosin	1.00^e-18^	89

Early Larval	821	1349	18	7.46^e-05^	Troponin C	1.00^e-38^	70

Late Larval	412	975	80	3.10^e-22^	Ribosomal Protein L31	1.00^e-51^	83

^1 ^R-statistic was based on equations in Stekel et al. (2000) and compared relative abundance of each libraries prevalence in contiguous sequences from all available sequences.

^2 ^*p*-values were calculated to establish appropriate significance thresholds to confirm individual library abundance. *p*-value is in reference to significance of caste bias of singletons in contigs formed using a chi-squared test on singleton representation in each contig.

### *In Silico *Analysis Using Protein Prediction

The second *in silico *analysis of the EST libraries (Table 3) utilized the protein function predictor [18]. Proteins associated with anti-bacterial humoral response and translational elongation were two protein functions predicted for contigs with a transcript bias in the soldier cDNA library (Table 3). Putative functions of neurotransmitter secretion or cholesterol absorption (Figure 5C) as well as cell adhesion, and hydrolase activity (not shown) were overrepresented in the alate cDNA library. Predicted protein functions of protein synthesis and ligase activity (Figure 5D) as well as regulation of transcription, metabolism, stress response, cell adhesion, and spermatogenesis (not shown) exhibited a transcript bias in the late larval stage cDNA library.

**Table 3:** *In silico *analysis and predicted protein function of 10 contigs with the highest R-value with no putative role assigned by a BLASTX search.

Library Bias	Contig #	Length (bp)	**R**^**1**^	***p*-value**^**2**^	**Predicted Protein Function**^**3**^
Soldier	910	1255	15	6.92^e-11^	Antibacterial humoral response; Translation elongation

Alate	545	1346	382	6.39^e-128^	Neurotransmitter secretion; Cholesterol absorption

Alate	142	1745	76	1.54^e-25^	Cell adhesion; Hydrolase activity

Late Larval	366	478	97	5.45^e-70^	Protein biosynthesis; Ligase activity

Late Larval	598	903	49	2.00^e-19^	Regulation of transcription; Metabolism

Late Larval	516	393	39	2.04^e-16^	Stress Response

Late Larval	533	474	31	7.12^e-13^	Cell adhesion

Late Larval	582	1133	26	9.43^e-09^	Spermatogenesis; Cell-cell adhesion

Late Larval	560	197	24	3.89^e-09^	Ribosomal protein-nucleus import

Late Larval	596	747	14	1.27^e-07^	Amino acid metabolism

^1 ^R-statistic was based on equations in Stekel et al. (2000) and compared relative abundance of each libraries prevalence in contiguous sequences from all available sequences.

^2 ^*p*-values were calculated to establish appropriate significance thresholds to confirm individual library abundance. *p*-value is in reference to significance of caste bias of singletons in contigs formed using a chi-squared test on singleton representation in each contig.

^3 ^Putative protein functions were predicted using: http://dragon.bio.purdue.edu/pfp.

## Discussion

### General Library Analysis

The gene discovery rate of sequences for each library ranged from 47% to 60%. The high proportion of genes with no known function in this analysis is likely due to the paucity of termite genes in public databases. In addition, it is possible that some of the unidentified genes may be of protozoan origin because we did not explicitly attempt to exclude protozoa as we prepared the mRNA for cDNA library construction. Finally, some of the sequenced fragments may have been 5' or 3' UTRs or other non-coding regions although a BLAST search for UTRs did not result in an unusually large number of significant hits. In this study, about 12% of the sequences in the worker and late larval libraries were associated with ribosomal function. A previous study [19] on *R. flavipes *immatures and adult reproductive castes also found rRNA-like gene expression in workers.

The GO analysis indicated that the proportion and variety of expressed genes in each of the three castes were similar to one another and there were minor differences among the three castes and the two larval stages. In the analysis involving molecular function terms, the late larval cDNA library exhibited the fewest number of classes of genes. Although this could be a true reflection of differential gene expression patterns, it is possible that the pattern could have resulted due to sequencing the fewest number of clones from the late larval cDNA library relative to the other four libraries. The proportion and variety of genes in the analysis involving biological processes was largely similar among the five cDNA libraries. The fact that most of the GO term representation patterns were similar among the five cDNA libraries suggests that caste-specific differences may involve both transcriptional and post-transcriptional regulation of genes.

In four of the five cDNA libraries (with the exception of late larval library), several genes putatively associated with reproductive functions were expressed. On the surface, the observation that genes associated with reproduction are expressed in individuals of sterile castes such as workers and soldiers may seem counterintuitive. However, the expression of genes involved in reproductive functions in sterile castes is likely a reflection of the fact that *R. flavipes *workers have the ability to become third-form reproductives in case of the death of the primary or second-form reproductives [2]. The fact that some of reproductive function genes are also expressed in soldiers, which do not become third-form reproductives, suggests that the genes implicated in reproduction may have other functions unrelated reproduction. Some of the genes involved in reproductive function may be subject to post-transcriptional or post-translational modifications that may render their phenotypic effects undetectable in certain castes or life stages.

### *In Silico *Analysis

The most bias from the first grouping of sequences used for the *in silico *analysis was composed mostly of soldier singletons which matched a viral protein, the source of which is not clear. It is possible that the soldiers were infected with a virus or there was viral contamination in the soldier library; alternatively, soldiers could have a protein similar to the viral protein that is expressed at a higher level than in the other castes. This issue needs to be further explored using quantitative real time PCR of individuals from multiple colonies.

Two transcripts that were over-represented in soldier and alate cDNA libraries matched a protein that was similar to the *pebIII *ejaculatory bulb-specific protein in *D. melanogaster*. Previous studies suggest that this protein is expressed in pheromone glands of male flies and is similar to an odorant-binding protein in *Mamestra brassica *that has been implicated in sexual attraction and repulsion [20]; however, recent studies have also reported the presence of this protein in fly heads [21], the female reproductive tract [22], and in response to viral challenge [23]. The *pebIII *matches a chemosensory protein in *A. mellifera *(AmelCSP3); however, the expression pattern of this protein in a Northern blot analysis indicated a function in cuticle maturation [24]. The termite sequence was 55% identical to that of *D. melanogaster *(REFSEQ:NP_524966.1; e-value: 2.00e^-28^) and 52% identical to the *A. mellifera *sequence (e-value: 2.00e^-26^). It is unknown what role these proteins play in termites, but they are about 97% similar to each other, with a larger proportion of the transcripts being of alate origin, suggesting the presence of a greater number of transcripts in alates relative to soldiers.

Three other genes showed a significant transcript bias in the alate library. The first gene was most similar to the mitochondrial cytochrome c oxidase subunit. Higher levels of cytochrome c oxidase expression were previously discovered in dealate ants and hypothesized to be involved in programmed wing cell death [25]. The second gene matched the cellulase b gene in the bacterium, *Cellulomonas firmi; *the prevalence of this in alates may reflect higher levels of symbiont load in alates relative to other castes as the gene is of bacterial origin. Alates need extra stores of symbionts to establish the bacteria in the guts of workers and soldiers in a nascent colony to assist in cellulose degradation [3]. The final gene matched an ATP synthase subunit suggestive of higher initial energetic needs of colony founders.

The remaining genes analyzed showed biases primarily in the early larval stage library. Most of the genes that matched are involved with general energy production and muscle contraction [26]. It is not clear whether these higher need for energetic reserves and muscle contraction represent the general colony tasks that the larvae perform relative to the more specialized tasks typically undertaken by the older workers and soldiers.

The second group of genes subjected to the R-statistic analysis was of those that showed a library bias of singleton composition but did not return a significant BLAST hit; genes in this group were nonetheless submitted for a putative function prediction based on the protein translation. Our results suggested that soldiers have a higher level of expression of two genes putatively involved in immune function, whereas in other eusocial insects, the pattern of increased immune investment was generally seen in the reproductive individuals [27]. It is important to consider that this sequence was on the lower end of the R-statistic acceptance levels, and thereby its representation was not as biased for one caste or the other relative to those with higher R-statistic values.

The two transcripts with an over-representation in the alate library included proteins putatively involved in neurotransmitter secretion or cholesterol absorption (contig 545), cell adhesion, and hydrolase activity. The over-expression of these genes may indicate a higher level of investment by alates in brain and cell-to-cell communication which may be helpful in mate finding, mate recognition, and colony establishment. Many of the transcripts from late larval stage library have a function putatively associated with energetic needs and metabolism.

Finally, previous studies have implicated two hexamerins (*Hex-I *and *Hex-II*) in soldier-worker differentiation in *R. flavipes *[28,29]. We recovered both hexamerin I and II in worker, alate, and early larval stage libraries and hexamerin-I in the soldier library; however they did not exhibit differential expression in the *in silico *analyses, most likely because we did not examine the transitional form between soldiers and workers, as was done in the previous studies [28,29].

## Conclusions

We presented an analysis of 15,351 randomly chosen cDNA fragments from 5 different cDNA libraries and identified approximately 5,000 ESTs. Relative to the honey bees, little is known about the gene expression differences among various castes and life stages. Therefore, the compiled EST data base will be a valuable resource for future studies on the sociogenomics of termites in general and *R. flavipes *in particular. In addition, the *in silico *analysis revealed several patterns that could be the subject of future functional genomics studies. For example, the caste-biased transcript abundance of several genes indicates that these proteins may be playing specialized roles in each of the castes or developmental stages. The presence of proteins putatively involved in reproduction in sterile castes is intriguing. It is possible that these proteins play multiple roles or they may be subject to post-transcriptional or post-translational modifications.

## Abbreviations

**cDNA: **complementary deoxyribonucleic acid; **GO: **Gene ontology; **rRNA: **ribosomal ribonucleic acid.

## Authors' contributions

SK conceived the project; SK and MS undertook laboratory work and data analyses; DC helped with data analysis; SK wrote the paper with help from MS. All authors read and approved the final manuscript.
